# Developing an Optical Image-Based Method for Bridge Deformation Measurement Considering Camera Motion

**DOI:** 10.3390/s18092754

**Published:** 2018-08-21

**Authors:** Vahid Abolhasannejad, Xiaoming Huang, Nader Namazi

**Affiliations:** 1 School of Transportation, Southeast University, Nanjing 211189, China; huangxm@seu.edu.cn; 2 Department of Electrical Engineering and Computer Science, The Catholic University of America, Washington, DC 20064, USA; namazi@cua.edu

**Keywords:** bridge deformation, motion correction, image-based technique

## Abstract

Since deformation estimation may lead to errors occurring when the camera vibrates, it is necessary to remove the image global motion before computing real bridge deformation. In this study, a combination of image motion correction algorithm and 2D image-based deformation measurement technique was utilized to address the issue of camera motion during the image data acquisition for bridge deformation measurement. Based on the proposed methodology, the image motion parameters were estimated by defining an effective sub-image in the image and using Iterative Affine Motion Estimator. Then the estimated parameters were applied to all pixels of each captured image to remove the motion effect. Finally, the corrected images were used to analyze by a 2D image-based deformation measurement technique in order to extract and measure real bridge deformation by tracking artificial or natural targets. The proposed methodology was validated by two experiments in the lab and field environments. Achieved results show the accuracy and reliability of the proposed methodology.

## 1. Introduction

Deformation measurement is a key factor for safety and serviceability evaluation of bridge structures during their service life [1]. To assure safe bridge performance, deformation must remain within acceptable limits over the entire life of the structure. Therefore, it is crucial to accurately and efficiently estimate and monitor the deformation amplitude of bridges in order to prevent unpredictable collapse and deterioration. There are two major methodological categories for deformation estimation of bridges; namely contact-based and non-contact-based.

The contact-based measurement methods can be implemented through conventional measurement devices including dial gauges, Linear Variable Differential Transformers (LVDTs), and accelerometers [2,3,4]. Dial gauges and LVDTs are accurate devices for deformation measurement, however, they need to be fixed, i.e., attached to the measurement point of the structure. Additionally, using this method deformation can be measured in one direction only. Accelerometers obtain the deformation through an indirect procedure that makes them less accurate and more sensitive than other traditional instruments. These three devices also need voluminous instruments that must be installed on the structure through a challenging and difficult procedure. As a result, these instruments are at high risk for damage during the data gathering process.

Non-contact-based measurement approaches use high technology instruments in order to overcome the drawbacks of the conventional deformation sensors. Laser Doppler Vibrometer (LDV) [5,6], Global Positioning System (GPS) [7,8,9,10,11,12], laser scanning [13,14,15], and image-based measurement methods [16,17,18,19,20,21,22,23,24,25] are among the most popular non-contact devices. However, all of these methods suffer from drawbacks that limit their application for deformation measurement of bridges. For example, despite the fact that LDV is a powerful technique for deformation measurement, the high cost of this system prevents its wide application. In addition, the laser intensity makes this method dangerous to use, especially over great distances. Reduced accuracy and sampling rate are the limitations of GPS for bridge deformation measurement; however, its precision can be affected by the number of existing satellites. As an optical remote sensing method, the laser-scanning system is able to make the 3D cloud point of an object. This system does not need any target or other kinds of surface preparation for measurement. In addition, since the data capturing is a sequential procedure, this method is suitable for deformation measurement while the static load is applied.

Among all the aforementioned non-contact measurement methods, the image-based method may be more applicable for the following reasons:

(1) Remote deformation measurement of bridges within the range of a few meters to hundreds of meters.
(2) Fully automated with performance in a real time manner.

Non-contact image-based measurement methods have been widely used recently for deformation measurement of road infrastructures, especially for bridge deformation measurements. Based on the study conducted by Lee and Shinozuka [26] image processing methods were applied to measure bridge dynamic deformation in real-time. In this study, a camcorder was used to capture the image data. In addition, the displacement measurement results by the proposed method agreed with those obtained through the laser vibrometer with small measurement noise. Digital image correlation was utilized by Yoneyama [27] to measure bridge deformation. One of the limitations of this method is the need to use the speckle pattern on the structure surface. Moreover, high quality and expensive cameras are needed to obtain reliable results. Jiang et al. [28] presented a literature review to describe the previously research works related to bridge deformation and structural monitoring. In this research, the photogrammetric methods of bridge measurement have been investigated since 1970s and the evolution of cameras, targets, network control, and software were described. A close-range photogrammetric bridge deformation measurement system was developed by Jiang and Jáuregui [29]. This system was applied to measure bridge deformation under static loading conditions. Various kinds of camera configurations were installed and the methodology was validated by experiments in the laboratory and field environments. In a study by Valença et al. [30], deformation of a footbridge subjected to different loading situations was monitored. The results showed that photogrammetry is advantageous comparing to traditional methods in structural monitoring. In other research conducted by Riveiro et al., a low cost photogrammetric method was utilized to measure the vertical bridge under-clearance [18]. Through this procedure, bridge elements were reconstructed in a 3D space. The advantage of this method is to perform routine bridge inspection, affording accurate and safe measurement. An image-based methodology was developed by Ribeiro et al. [31] for the estimation of dynamic railway bridge deformation. Equipment utilized consisted of high speed camera with a frame rate of 64 fps to 500 fps, camera lens, targets, and lamps installed for measurement. Also, laser sensing technology was utilized for bridge deformation measurement by Zhao et al. [32]. The results of that study showed the precision of the proposed deformation measurement system. The vertical deformation of bridges was measured in a real-time, non-contact, target-less manner by a research study in which the off-axis digital image correlation method was utilized [33].

The research presented thus far indicates that non-contact image-based techniques are superior to other methods; however existing techniques do bear some limitations. As can be seen in most of the image-based deformation measurement methods, a fixed-position camera was utilized to measure the bridge deformation. In the field, camera motion due to ambient conditions such as wind results in motion noise which affects measurements, leading to significant error in the deformation measurements. In some field measurement cases, it was suggested that the probable camera movement may be somewhat decreased by installing windshield equipment on the camera at the bridge site [26].

It is also noted that some authors have reported the use of reference points for estimating camera motion relative to a movable camera. For example, Olaszek [34] developed an optical image-based method in which a reference system was defined through one separate target and camera channel in order to estimate the probable movement of the camera and correct results obtained by the vibrated camera in the real field bridge deformation measurement. However, using this reference point requires additional equipment such as camera and targets. Moreover, using a reference-point system limits the number of monitored points which depends on the number of cameras and targets utilized. Consequently, the use of a reference-point is limited in scope and does not efficiently address the issues associated with ambient camera motion. Wagner et al. [35] also proposed a bridge monitoring method using a video-tacheometer or Image Assisted Total Station (IATS) to determine the absolute bridge displacement. Through this system, photogrammetric image measurement methods are utilized to detect the target points and total station methods such as precise angle and distance measurement (to prisms or reflectorless) are employed to control the stability of the monitoring station. In another study conducted in 2015 [36], the usage of IATS was investigated to monitor the vibration and displacement of civil engineering structures. In this study, the possible rotations of the camera (due to rotations of telescope or tilts of the IATS) were corrected by using total station to achieve the accurate displacement values. In this case, additional sensors and data acquisition system are required to estimate the possible movement of camera. Data fusion techniques should also be adopted to integrate different data sources. These limitations, along with the low camera frame rate make a challenging and costly procedure for measurement of bridge deformation. Considering these facts, it is necessary to develop a methodology which is able to address the issue of camera motion before extracting the real deformation of bridge structure, one which is convenient, time-saving and cost effective.

As such, the current study is devoted to the development, implementation and presentation of a non-contact image-based method for the measurement of bridge deformation considering camera motion. The proposed method deals with ambient camera motion without requiring any additional optical equipment or extra time and cost for arranging the measurement procedure. By the proposed method, the image global motion caused by the camera motion is estimated and the image is corrected before calculation of real bridge deformation. Therefore, first the image motion correction method is presented. Then the 2D image-based deformation measurement method is utilized to measure the real bridge deformation by tracking artificial or natural targets. Thirdly, the proposed methodology is validated through asphalt Uniaxial Compression (UC) test in the lab and finally the validated methodology is tested and verified on a real bridge in the field.

In the remainder of this work, the principles of the algorithm for image motion correction as well as the 2D image-based deformation measurement technique are described. Then, the experimental study in the lab and the field is conducted to verify the proposed methodology. Finally, the future research challenges are presented.

## 2. Methodology of Deformation Measurement

**Figure 1:**
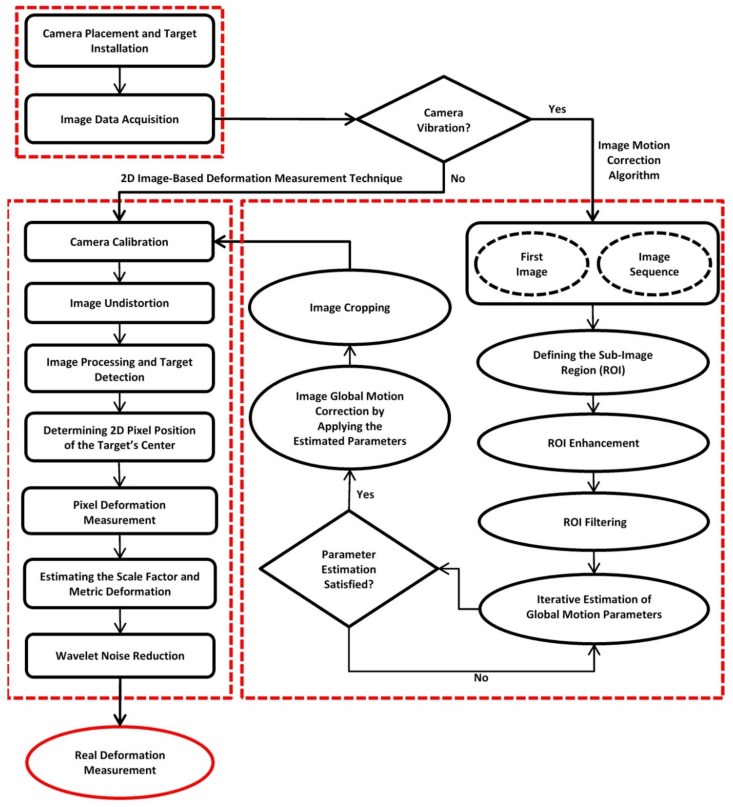
Schematic diagram of 2D image-based motion correction methodology.

In this section, the methodology of deformation measurement under described conditions is presented. The proposed approach addresses two main challenges, camera motion due to ambient conditions such as wind in the field environment as well as accurate measurement of bridge deformation through a non-contact image-based method. The proposed methodology is a combination of image global motion correction algorithm and 2D image-based deformation measurement technique. This combination is generally called “2D image-based motion correction methodology” in this paper. Figure 1 illustrates the schematic diagram of 2D image-based motion correction methodology. In the following, each part of the methodology is described.

### 2.1. Image Global Motion Correction Algorithm

In this section, an effective technique for the correction of the image global motion is described. Several research works have been conducted for image global motion estimation and correction [37,38,39,40,41]. Generally, there are two types of pixel motion in an image, namely global and local motion. Global motion includes the translation and/or rotation of the image due to camera motion. Local motion generally is due to the object’s movement within the image area, such as real bridge deformation. In order to measure accurate local motion, one should separate local motion from the image global motion which occurs as a result of camera motion. First a square sub-image, including the fixed natural distinctive features but not including the parts with local motion, is considered as the reference region of interest (ROI) in the image sequence for the estimation of image global motion parameters. This square sub-image is defined and selected from within the image so that it is only globally affected by motion and distinctive fixed elements exist within the defined region. In this study, the size of ROI is considered equal to 256 × 256 pixels. It should be noted that the natural background features (within the ROI) are assumed to be stationary. These features may be natural such as buildings, rocks, trees, etc. or artificial. The image motion correction procedure is started by applying image enhancement and filtering on the ROI using the Adaptive Wiener Filter (AWF) and blurring filter [42]. Then, in order to estimate and correct the image global motion, the Iterative Affine Motion Estimator (IAME) is utilized. Refer to Namazi et al. [39] provides an informative explanation of the concept of IAME. Through this algorithm, the ROI intensity equations in two successive images are:

(1) $$H_{1}\left(x\right)=I\left(x\right)+n_{1}\left(x\right)$$

(2) $$H_{2}(x)\;=\;I\left(x-P_{g}\left(x\right)\right)+n_{2}\left(x\right);\;x\in {\;\Gamma }_{g}$$

where $x={\left(x_{1},x_{2}\right)}^{T}$ is the vector of pixel coordinate, I(x) is the noise-free intensity of the previous ROI, $H_{1}\left(x\right)$ is the noisy intensity of the previous ROI and $n_{1}\left(x\right)$ represents noise, $P_{g}\left(x\right)$ specifies the global motion vector at the location x, and $n_{2}\left(x\right)$ denotes noise. In addition, the region $\Gamma_{g}$ is the respective partition of $H_{2}\left(x\right)$ corresponding to globally displaced pixels [39].

In this algorithm, the first image is considered as the reference image and the image sequence are continuously (frame by frame) analyzed to estimate the global motion parameters and remove the effect of camera motion. So, by following the IAME algorithm, the image global motion parameters, including translation, rotation, expansion, zooming, skewing and scaling are estimated. It means that the proposed image motion correction algorithm is capable of estimating and removing the camera translation and rotation in different camera axes. The final estimated global motion parameters obtained through analysis of ROI are then applied on the all image pixels; subsequently each image is corrected considering the image global motion.

With all the captured images globally corrected, they were cropped from a fixed reference point at the bottom left of each image, thus preparing all the images for deformation measurement analysis. This fixed reference point could be selected by determining a crop point from among the existing elements in the image.

### 2.2. 2D Image-Based Deformation Measurement Technique

After all the obtained image data were globally corrected, the 2D image-based deformation measurement technique was applied to calculate the real bridge deformation. The proposed image-based technique is a non-contact target-based method which is described below.

First the intrinsic and extrinsic camera parameters are calculated through MATLAB Camera Calibration Toolbox [43]. These parameters can be shown as the following Equation [44]:

(3) $$K=\left[\begin{array}{ccc} \alpha_{u} & s & u_{0}\\ 0 & \alpha_{v} & v_{0}\\ 0 & 0 & 1 \end{array}\right]$$

where $\alpha_{u}$ and $\alpha_{v}$ are the scale factor along the u and v coordinate axes, respectively; c indicates the image coordinates of the principal point, ${\left[u_{0},v_{0}\right]}^{T}$; and s is the skew.

In the next stage, the distorted image is converted into an undistorted image by applying the camera calibration results [45]. With the radial and tangential distortion functions known, and using the following equations, the distorted coordinates are calculated.

**Figure 2:**
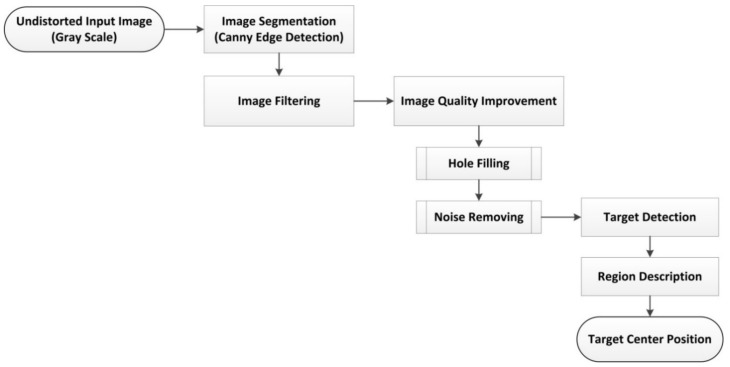
Flowchart of the image processing and analysis algorithm.

Then an image processing and analysis algorithm is utilized to detect the target points including artificial or natural targets and estimate the center position of targets. It should be noted that in some situations, access to the monitored bridge and installation of artificial targets is difficult. So, the natural targets are installed and tracked through the image processing and analysis algorithm to reduce the required cost and time of bridge deformation measurement. The detailed flowchart of image processing and analysis algorithm is shown in Figure 2.

As shown in Figure 2, the undistorted image as the input image is being processed through an image processing algorithm. First the canny edge detection algorithm is applied to detect the objects boundaries [46]. Then the effective area around the target is defined by using the filtering algorithm. The mentioned target can be artificial (for example, circular) or natural feature which is located within the defined effective area or region of interest (ROI). In the next stage, by filling the holes in the image and removing noises, the target is detected [47]. Finally, the 2D center position of each target is determined by the image analysis algorithm of region descriptor [48].

Accordingly, all the successive images are considered to be analyzed for estimation of momentary metric position of target center at time t. Having the center pixel position of each target in the captured image at time (t+1) and (t) that correspond to $\left(x_{t+1},y_{t+1}\right)$ and $\left(x_{t},y_{t}\right)$ and the image scale factor (p), the metric position of each target center is calculated through the following equation:

(4) $$Y_{t+1}=Y_{t}+p\left(y_{t+1}-y_{t}\right)$$

where $Y_{t}$ is the metric vertical position of each target center at time (t) and $Y_{t+1}$ is the metric vertical position of each target center at time (t+1). The initial value of $Y_{t}$ is considered equal to zero. The image scale factor can be calculated by comparing the object dimension in the real field and in the image [49].

Finally, the wavelet transform is utilized to reduce the unexpected noise or variations of the obtained results [50]. By applying this de-noising procedure, more smooth results will be achieved.

## 3. Experimental Study

In this section, the proposed 2D image-based motion correction methodology is tested in both lab and field environments to verify the capability of this method for addressing the issue of camera motion during image data gathering. First, asphalt Uniaxial Compression (UC) test is performed in the lab to validate the proposed methodology. In this experiment, a cylindrical asphalt specimen is loaded by the Universal Testing Machine (UTM) and deformation is measured and computed by both traditional and 2D image-based motion correction methods. Then the proposed methodology is applied on the real bridge in the field environment to show the reliability of the proposed methodology.

### 3.1. Lab Experiment

#### 3.1.1. Test Specifications and Data Preparation

**Figure 3:**
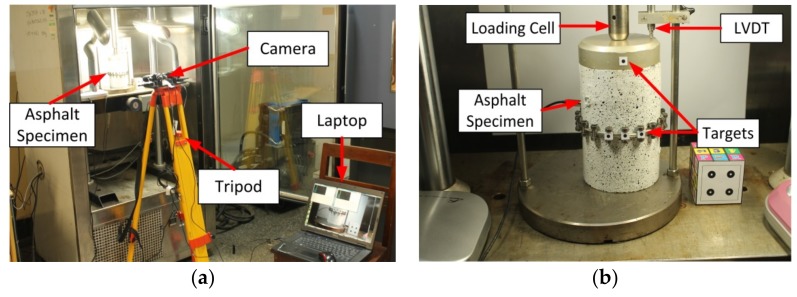
Test configuration: (**a**) test set up; (**b**) asphalt specimen.

In order to validate the proposed methodology in the laboratory, a test was performed on the asphalt specimen. This Uniaxial Compression (UC) test had been carried out by Sun et al. in 2016 [23]. In this test, vertical deformation occurs by applying the compressive force on the cylindrical sample. This deformation was measured through 2D image-based motion correction methodology and validated by using conventional methods including LVDT. First, we briefly present the configuration and specifications of the UC test as shown in Figure 3.

**Figure 4:**
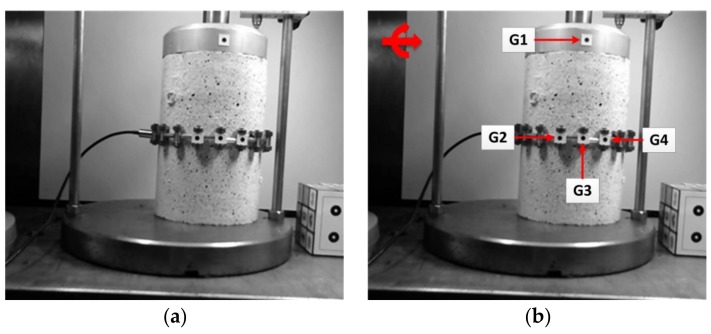
Image data preparation: (**a**) main captured image; (**b**) synthetically moved image.

In this experiment, a cylindrical asphalt specimen with a height of 15 cm and a diameter of 10 cm was loaded under the variable vertical load, which resulted in vertical deformation in the specimen during the time of loading. This actual deformation of the specimen was measured using the LVDT installed at the top of the specimen. The time and applied load were also measured by the Universal Testing Machine (UTM) during the test. In addition, to estimate the vertical deformation through the 2D image-based motion correction methodology, four circular black targets with a 4 mm diameter $\left(G_{1},{\;G}_{2},{\;G}_{3},{\;G}_{4}\right)$ were used and installed at specific points on the surface of the asphalt specimen as shown in Figure 4b. That made it possible to estimate the deformations at these target points using image data processing and analysis algorithm. The camera used in this test was a webcam (Logitech C270 HD, Suzhou, China) with a resolution of 1280 × 960 pixels and a maximum frame rate of 30 fps.

The test image data and the real data from LVDT were gathered during the loading operation. The calibration image data was also collected before starting the main test. Also all image data captured by the camera is transferred to and stored on a hard drive.

However, in the research work conducted by Sun et al. [23], it was assumed that the camera was fixed and remained motionless throughout the entire test. In this situation, the vertical deformation of asphalt specimen will be estimated directly by 2D image-based deformation measurement technique. But, in the present study, the researchers aim to calculate vertical deformation taking into consideration camera motion. Therefore, in order to take into account the effect of camera motion during the image data collection, the images of asphalt specimen which were captured through a fixed camera were synthetically translated (along X and Y axis of image) and rotated (about the normal axis of image plane) up to a few pixels and degrees in each frame through a random procedure to simulate the real camera motion. Figure 4 shows the main captured image of asphalt specimen which was considered for testing the 2D image-based motion correction methodology. As can be seen, the original image as well as the synthetically moved image is shown in this figure.

#### 3.1.2. Image Data Analysis

Three types of image data were generated: original image data obtained through fixed camera (Group 1), image data generated by synthetically image translation and rotation or before applying the image motion correction algorithm (Group 2), and corrected image data after applying the image motion correction algorithm (Group 3).

As for the data in the first group, it is possible to directly use the 2D image-based deformation measurement technique to calculate the asphalt vertical deformation. So, firstly, the original captured image data were undistorted considering the camera calibration parameters as mentioned in Table 1.

**Figure 5:**
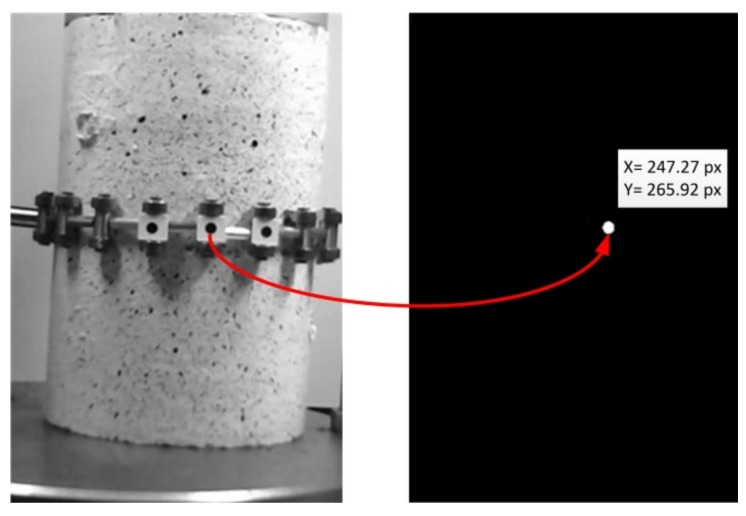
Target detection and positioning.

Then the images were processed and analyzed in order to detect the target and determine the pixel position of each target center during the time. The circular target detected by applying the image processing and analysis algorithm is shown in Figure 5.

Having the pixel locations of the target center in each image sequence, as well as the image scale factor, the asphalt vertical deformations were computed. Subsequently the metric positions of each target center during the time were estimated using Equation (4). It should be noted that in this experiment, it is assumed that the diameter of a circular target is considered as the known physical length on the asphalt surface in order to estimate the scale factor. Finally, the wavelet transform was used to reduce the existing noise of the results.

For the analysis of the image data in Group 2, the same method as the first group was applied to calculate the asphalt vertical deformation. Since the images were translated and rotated in this state; thus the achieved deformation results were not be going to be reliable. If the camera vibrated due to ambient conditions, direct use of the 2D image-based deformation measurement technique would not be effective to achieve reliable results. Therefore, in order to solve the problem, first it is necessary to correct the image data through the image global motion correction algorithm as described in the previous section and then apply the 2D image-based deformation measurement technique to estimate the vertical deformation.

**Figure 6:**
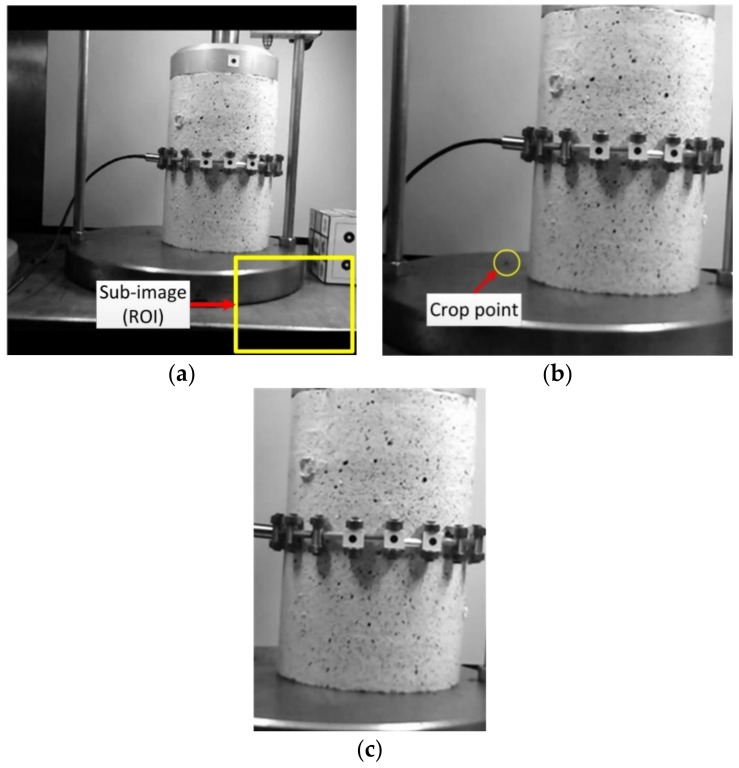
Image motion correction procedure: (**a**) ROI definition on a synthetically moved image; (**b**) image correction and determining a crop point; (**c**) Cropped corrected image as the final output image.

As such, the image motion correction parameters were first estimated by taking into account a sub-image called ROI of the main image. Subsequently the obtained parameters were applied on all pixels of the entire image, as shown in Figure 6a,b. In this way, the images were corrected with occurred movements removed. Finally, the corrected images were cropped by selecting a natural point located at the bottom left side of the image (Figure 6c). As such, the corrected images (Group 3) were generated. Therefore, these images were considered as the final output for applying the 2D image-based deformation measurement technique as described above.

**Figure 7:**
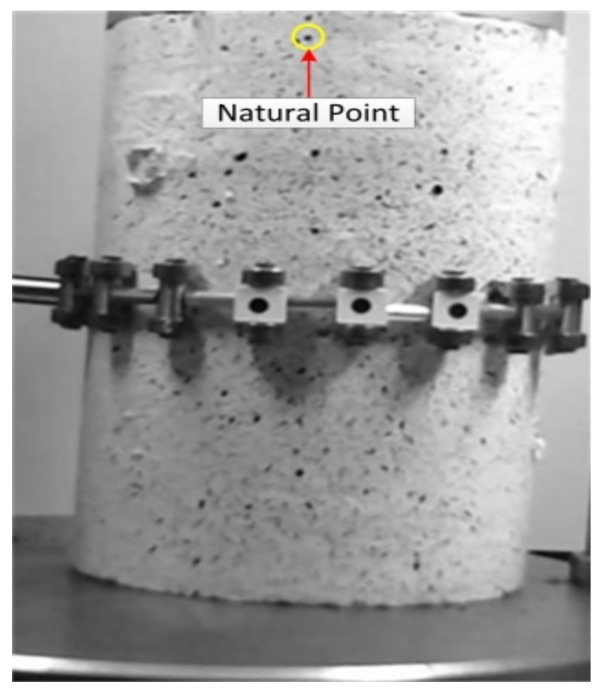
Natural point instead of target $G_{1}.$

Finally, by applying the proposed methodology, three categories of results are achieved from three groups of images, including original images, synthetically moved images (before image motion correction) and corrected images (after image motion correction). Then, by comparing the achieved deformation results from analysis of all image data groups to each other and with those obtained through LVDT, the proposed methodology, including the image global motion correction algorithm and the 2D image-based deformation measurement technique were validated. It should be noted that the deformation results of LVDT are only available at the location of target $G_{1}$. As can be seen in Figure 7, since target $G_{1}$ can not be detected and analyzed in the image, a natural point at the top of the asphalt specimen near target $G_{1}$ was considered to have been tracked. Detecting and tracking the natural target is performed by using the proposed image processing and analysis algorithm. It should be noted that all the stages of the proposed 2D image-based motion correction methodology including the image motion correction algorithm and 2D image-based deformation measurement technique is accomplished through MATLAB software.

#### 3.1.3. Comparison of Results and Methodology Validation

**Figure 8:**
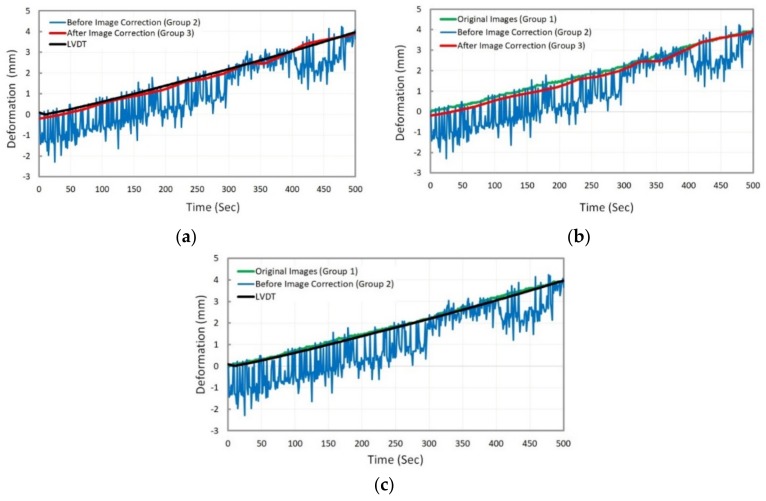
Comparison of deformation results at target $G_{1}$ (or natural point) by analysis of three image data groups.

Based on the proposed methodology, the image data (Groups 1, 2 and 3) were analyzed and the comparison was accomplished by comparing the deformation results of asphalt specimen through analysis of the three groups of image data previously described. First, these results are compared at target $G_{1}$ or natural point, which is located at the top of the asphalt specimen. The comparison of deformation results versus the time are illustrated in Figure 8.

In Figure 8 above, the deformation results were compared considering three different states:

(a) deformation measurement using Group 2, Group 3, and LVDT,
(b) deformation measurement using Group 1, Group 2, and Group 3,
(c) deformation measurement using Group 1, Group 2, and LVDT.

The comparison results showed that the deformation results obtained from the analysis of corrected images (after image motion correction) agree with those obtained from the analysis of original images or LVDT as the ground-truth value (Figure 8a,b). This could demonstrate the reliability of the proposed methodology, including the image motion correction algorithm and 2D image-based deformation measurement technique. In addition, the accuracy of 2D image-based deformation measurement technique was approved by comparing the results of the original images analysis and LVDT (Figure 8c). For better comparison, two measures of root mean square error (RMSE) and normalized root mean square error (NRMSE) were used to quantify the accuracy of both the image motion correction algorithm and the 2D image-based deformation measurement technique. These measures are defined in the following:

(5) $$\mathrm{RMSE}=\sqrt{\frac{1}{n}\overset{n}{\underset{t=1}{\sum }}{\left(E_{t}-O_{t}\right)}^{2}}$$

(6) $$\mathrm{NRMSE}=\frac{\mathrm{RMSE}}{O_{\max}-O_{\min}}=\frac{\sqrt{\frac{1}{n}\sum_{t=1}^{n}{\left(E_{t}-O_{t}\right)}^{2}}}{O_{\max}-O_{\min}}\times 100$$

where n is the number of measurement data; $E_{t}$ is the estimated deformation at time t (as measured by 2D image-based deformation measurement technique); $O_{t}$ is the true deformation value at time t (as measured by LVDT); and $O_{\max}$ = $\max\;(O_{t}$), $O_{\min}$ = $\min\;(O_{t}$).

The existing errors were calculated and summarized in Table 2 based on Equations (5) and (6) above. As can be seen in Table 2, the low measurement error (4.57%) shows the accuracy of the proposed methodology. In addition, the obtained results showed that the natural target can be effectively and accurately used instead of artificial target for deformation measurement.

**Figure 9:**
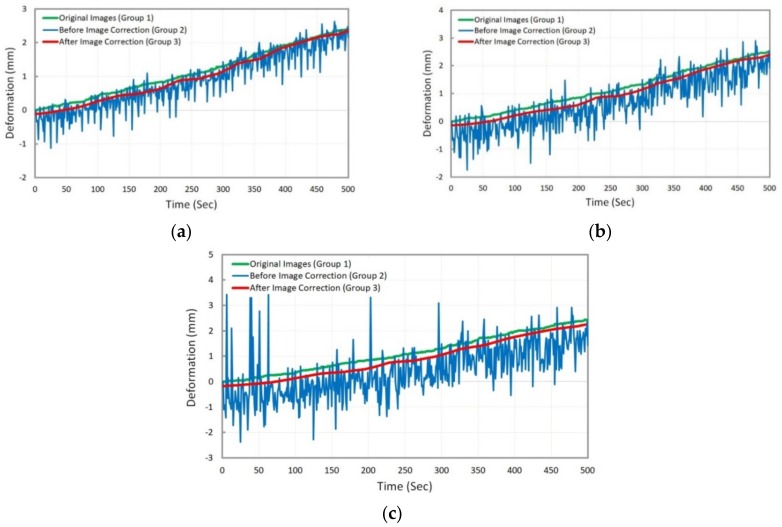
Comparison of deformation results: (**a**) at Target $G_{2}$; (**b**) at Target $G_{3}$; (**c**) at Target $G_{4}$.

After validating the proposed methodology by deformation measurement analysis at target $G_{1}$ (or natural point), the results comparison was done by applying the proposed methodology at the remaining targets of $G_{2}$, $G_{3}$, and $G_{4}$. This comparison includes comparing the deformation results obtained from the analysis of image data Groups 1, 2, and 3 which corresponds to original images, synthetically moved images (before image motion correction), and corrected images (after image motion correction), respectively. The results are shown in Figure 9.

According to Figure 9, the trend of deformation results obtained by the analysis of image Group 2 shows no agreement with those obtained through the analysis of original images (image Group 1). Thus, by applying the image motion correction algorithm and correcting the images in Group 2, then analyzing the corrected images (Group 3), accurate deformation results are obtained (according to the Group 3 graph). More analysis was done to quantify the existing measurement error as shown in Table 3. Based on measurement errors, it can be concluded that the proposed methodology is of acceptable accuracy.

After verification of the proposed methodology in the lab, the real bridge experiment in the field environment under the vibration condition was conducted in order to measure the dynamic deformation of bridge.

### 3.2. Bridge Experiment

#### 3.2.1. Test Configuration and Data Analysis

**Figure 10:**
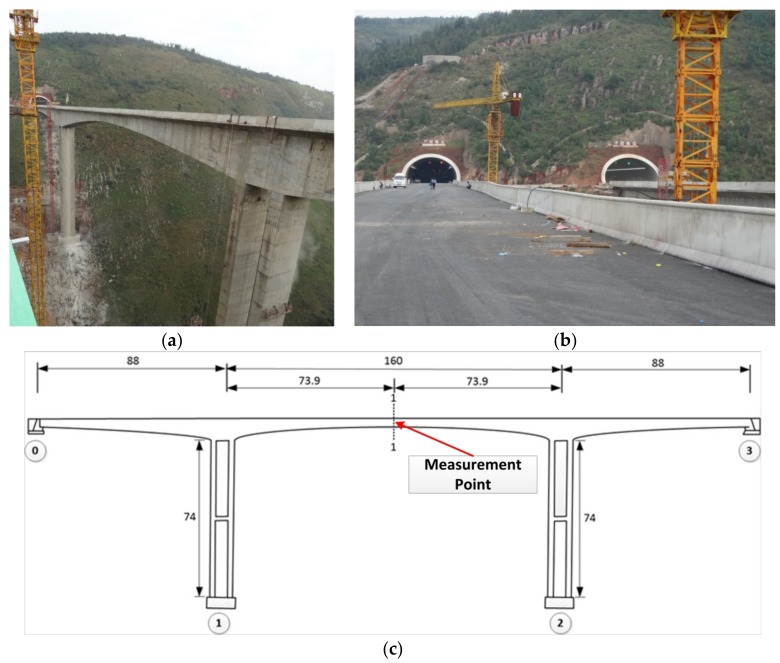
Bridge specifications (Measurement unit: m): (**a**) bridge structure; (**b**) bridge deck; (**c**) bridge dimensions.

In this section, a real bridge experiment in the field environment was conducted to investigate the reliability and efficiency of the proposed methodology. The test was performed at a new highway concrete bridge located in a mountainous area in Yunnan Province, China, with a total length of 336 m. It has three spans including one middle (length: 160 m) and two lateral spans (length: 88 m). Two separate parallel congruent unidirectional bridges span the valley to manage traffic flow coming from the opposing mountain. The bridge structure and its specifications are shown in Figure 10.

**Figure 11:**
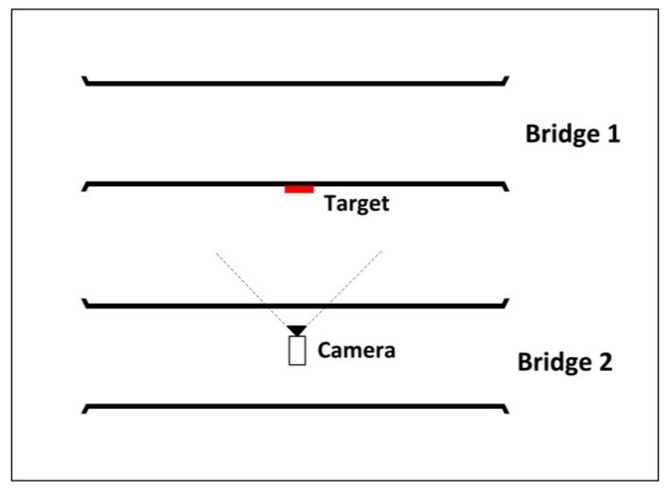
Configuration of image-based deformation measurement system.

As can be seen in Figure 10, the two parallel bridges are too high and the deformation measurement point is located at the middle point of the bridge. Considering the mentioned condition, the circular target (with diameter of 70 mm) was installed on the lateral surface of the first bridge and at the middle span and middle point. The camera (Sony DSC-HX300, Shenzhen, Guangdong, China) with the image resolution of 1440 × 1080 pixels and frame rate of 30 fps) was also placed on the second bridge (Figure 11). As such, the image data acquisition system was ready for image data gathering under dynamic load.

Since the camera was placed on the bridge deck, some ambient conditions such as bridge vibration caused by traffic flow or wind may affect the fixed position of the camera and subsequently, the camera may vibrate or move during image data gathering.

For bridge loading, a heavy cargo truck was passed over the bridge to apply the standard dynamic load. At the same time, the image data gathering was also performed during the bridge loading. Since the camera vibrated during the image collection procedure, the captured images were subjected to motion. In this case, the proposed 2D image-based motion correction methodology which was previously developed and verified was utilized to estimate and measure bridge vertical dynamic deformation considering image global motion.

**Figure 12:**
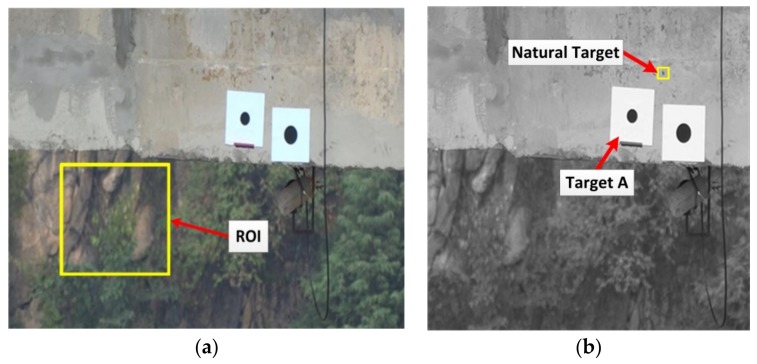
Image global motion correction: (**a**) specifying ROI for motion parameter estimation; (**b**) generated corrected image.

Therefore, the global motion of images was corrected using the image global motion correction algorithm described. The affine motion estimator was applied to the defined ROI in order to estimate the motion correction parameters. Subsequently, the whole image was corrected by applying these parameters to all pixels of the image as shown in Figure 12a. Then, the 2D image-based deformation measurement technique was utilized to estimate the bridge vertical dynamic deformation by tracking the circular target point A (see Figure 12b) in successive images.

In addition, a natural point near target A was defined to be detected and tracked as shown in Figure 12b to investigate the reliability of the proposed methodology. As can be seen in Figure 12b, an ROI was defined around the natural point and then the proposed 2D image-based deformation measurement technique was applied to track the natural target and measure the vertical deformation of bridge by analyzing the image data before and after image motion correction.

#### 3.2.2. Results and Discussion

**Figure 13:**
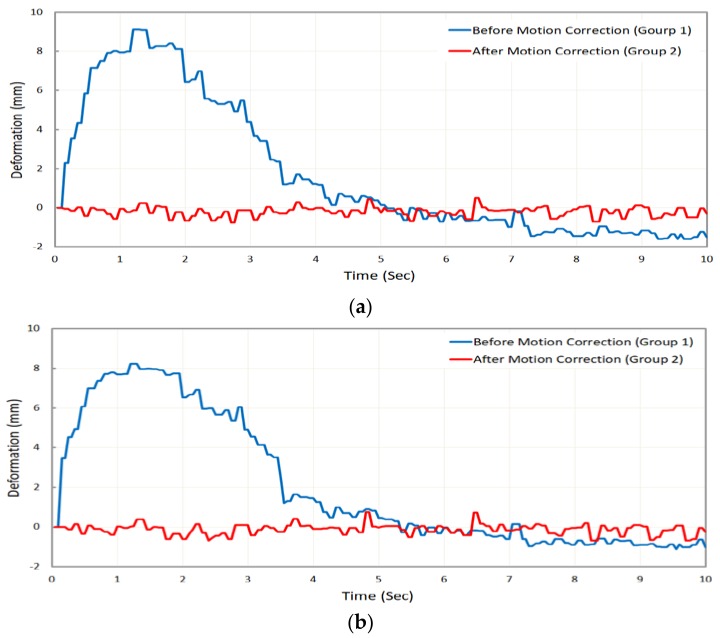
Comparison of bridge deformation measurement results before and after image motion correction: (**a**) at target A; (**b**) at natural target.

The captured images were corrected by analyzing the images obtained through the vibrated camera. Subsequently, the bridge deformation was calculated under dynamic load. In this experiment, two groups of images, those before and those after image global motion correction were analyzed. Figure 13 compares the results of bridge dynamic vertical deformation for each scenario at both target A and defined natural target. Comparison of the deformation results obtained by analysis of these two groups of image data enabled verification of the efficiency of the proposed methodology.

As indicated in Figure 13, the image global motion which occurred due to the camera motion during the data gathering was corrected, and finally the real bridge deformation was extracted. Based on the time-deformation diagrams in Figure 13, the obtained deformation results through analyzing and tracking circular target A and defined natural target showed the reliability of the proposed methodology.

Considering the experiments conducted in this study, it was proven that the real deformation of a bridge can be accurately calculated in the presence of camera motion. Compared to previous researches regarding bridge deformation measurement, it was found that the proposed methodology is a convenient, efficient and cost effective method for estimating bridge deformation in the presence of camera motion. The proposed methodology can be utilized as a primary step for addressing the problem of deformation measurement considering the camera motion.

While reliable results were achieved by the proposed method, some research issues can be investigated in the future which are explained in the following:

- According to the proposed method in this study, fixed background features are required to be defined for estimation of camera motion parameters. But finding stationary background features is challenging in some situations, for example on the shoreline. So, this challenge can be addressed by using inertial measurement unit (IMU) sensors in the Unmanned Aerial Vehicle (UAV) for camera motion estimation that can be investigated in the future.
- Different issues may affect the accuracy of bridge deformation measurement including: the distance between camera and bridge, using various types of cameras, and using different lenses. These issues can also be considered as the future researches.
- In this study, 2D image-based deformation measurement technique was developed to measure the in-plane vertical bridge deformation in presence of camera motion as described in Section 2. However, large out-of-plane deformation occurs in some of structures. For this case, the usage of multiple cameras such as stereo vision system would be an option to be investigated in the future.

## 4. Conclusions

The camera motion due to ambient conditions such as wind is a problem for accurate bridge deformation measurement, especially in the field environment. It is necessary, therefore, to find and develop an efficient image-based methodology to use the moved images as input and compute the real bridge deformation accurately and conveniently. This paper focused on the development and implementation of an image-based technique by which the issue of camera motion is addressed in less time with less cost. In addition, a combination of image motion correction algorithm and 2D image-based deformation measurement technique was developed to measure and extract real bridge deformation from the vibrated images in a continuous and convenient manner. Finally, two experiments were conducted (one in the lab and the other in the field) to test and verify the proposed methodology. The obtained images were categorized in different groups (original images, before image motion correction and after image motion correction), analyzed based on the proposed methodology through MATLAB. Then the real deformation was estimated for each image data group and compared. In addition, the validity of the proposed methodology was investigated by detecting and tracking artificial and natural targets. 

Comparing the deformation results obtained through analysis of image data before and after motion correction with those obtained through original images and LVDT verified the accuracy and efficiency of the proposed methodology. This methodology can be considered a suitable method for addressing the issue of camera motion.

Given the challenges addressed in this study through the proposed methodology including detection of fixed natural background features within the image to calculate camera motion parameters, removing camera motion (translation and rotation) by developed image motion correction algorithm, estimating the bridge displacement using one camera and 2D image-based deformation measurement technique, and measuring the vertical in-plane deformation of bridge structure, a monitoring base was made toward measuring the bridge deformation in presence of camera motion. In addition, the challenges which can be investigated in the future were presented, including the usage of UAV for measuring bridge displacement, adopting IMU sensors to estimate camera motion, and using multiple cameras for measuring out-of-plane motion of the structure. Considering the contributions of this study regarding the bridge deformation measurement in presence of camera motion, and the described possible future studies, it is expected that the capabilities of the proposed methodology can be extended and improved in the near future.

## Figures and Tables

**Table 1:** Camera calibration parameters.

Parameter	Value
Camera Focal Length	1428
Skew	γ = 0
Camera Principle Point	u_0_ = 604.66 v_0_ = 520.04
Radial Lens Distortion	k_1_ = −0.034 k_2_ = 0.053

**Table 2:** Deformation measurement error.

Comparison State	RMSE (mm)	NRMSE (%)
(a) Group 3-LVDT	0.12	3.06
(b) Group 1–Group 3	0.18	4.57
(c) Group 1-LVDT	0.91	2.31

**Table 3:** Deformation measurement error.

Comparison State	RMSE (mm)	NRMSE (%)
Target $G_{2}$ (Group 1–Group 3)	0.138	5.79
Target $G_{3}$ (Group 1–Group 3)	0.187	7.52
Target $G_{4}$ (Group 1–Group 3)	0.239	9.79
